# An anaerobic membrane bioreactor using a hollow fiber membrane and biogas agitation

**DOI:** 10.1016/j.mex.2020.101018

**Published:** 2020-08-02

**Authors:** Jin Sun, Yasunori Kosaki, Nobuhisa Watanabe

**Affiliations:** a Applied Chemistry, Environmental and Biomedical Engineering, Graduate School of Engineering, Osaka Institute of Technology, Ohmiya 5-16-1, Asahi-ku, Osaka 535-8585, Japan; b Department of Environmental Engineering, Osaka Institute of Technology, Ohmiya 5-16-1, Asahi-ku, Osaka 535-8585, Japan

**Keywords:** Biomethanation, Food waste, Anaerobic membrane bioreactor

## Abstract

Many biomass disposal demonstration projects are based on anaerobic digestion. However, the excessively slow anaerobic microorganism growth rate is a drawback because a decreased anaerobic microorganism population limits methane fermentation's efficiency. To ensure operation at higher loads, this study used an anaerobic membrane bioreactor (AnMBR) for maintaining anaerobic microorganisms’ growth, and this article introduces a series of improvements to address the reactor's shortcomings. Finally, we chose to mix-the internal biogas and conducted the experiment using a hollow fiber AnMBR.

• Introducing the design of a highly efficient and compact anaerobic membrane bioreactor (AnMBR).
• Introducing the initial OLR and the changes of HRT, SRT, TS, and flux of the permeate in the AnMBR after gradually increasing the load.
• Monitoring decomposition characteristics in the gas meter connection.

## Graphical abstract

**Figure:**
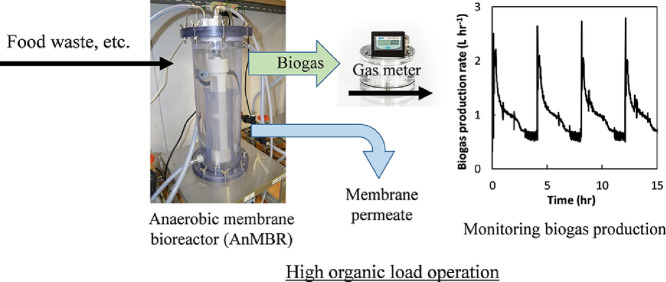

**Specifications table**

**Table:** 

Subject Area	Engineering
More specific subject area	Environmental Engineering
Name and reference of original method	The preparation method for the Artificial food waste (AFW) was based on the method used in previous research [1], [2], [3], [4].
	Design of the anaerobic membrane bioreactor system was taken from J. Sun et al [5,6]. 1. T. Konishi, T. Matsuoka, Y. Kosaki, Survey of food waste from university cafeteria considering biomass recycling and proposition of components for artificial food waste (in Japanese), Proc. Doboku Gakkai Kansai Shibu Annu. Conf. Heisei, 23th. (2011) VII-16–17. 2. K. Tanaka, Y. Kosaki, Study on improvement of methane fermentation using saccharification and ethanol fermentation as pretreatment (in Japanese), Annu. Conf. Japan Soc. Mater. Cycles Waste Manag. 26th. (2015) B7-7. 3. Y. Kosaki, Study on methane fermentation using biological ethanol fermentation as pretreatment (in Japanese), Doboku Gakkai Ronbunshuu. G 71 (7) (2015) III-47–III-55. 4. J. Sun, Y. Kosaki, N. Watanabe, M. Ishikawa, Production of methane-rich biogas and minimization of sludge by adopting ethanol fermentation for the pretreatment of biomethanation, J. Mater. Cycles Waste Manag. 21 (2019) 258–264. 5. J. Sun, Y. Kosaki, N. Watanabe, Higher load operation by adoption of ethanol fermentation pretreatment on methane fermentation of food waste, Bioresour. Technol. 297 (2020) 122475. https://10.1016/j.biortech.2019.122475. 6. J. Sun, Y. Kosaki, High operational loading by adoption of ethanol fermentation pretreatment for methane fermentation of food waste using an anaerobic membrane bioreactor, 3R International Scientific Conference on Material Cycles and Waste Management. 6th. (2020) C54.
Resource availability	N/A

**Method details**

## Introduction

Anaerobic digestion technology offers numerous economic and sustainability-related benefits. Anaerobic digestion recycles organic materials and turns them into biogas, which shows great promise for the production of large amounts of energy if appropriate technology is employed [7]. Once captured, biogas can generate heat and electricity for use in boilers and engines. Biogas can also be upgraded to biomethane and injected into natural gas pipelines or used as a fuel for vehicles.

However, anaerobic bacteria grow very slowly. A loss of bacteria also occurs when the processing load is increased by raising the feed volume, thus limiting the treatment of higher loads. Treatments utilizing the traditional methodology can only operate on a long hydraulic retention time (HRT). It is problematic to extend the solid retention time (SRT) and simultaneously shorten the HRT. Previous studies [8,9] have used the supernatant of centrifuged sludge as treated water and have returned the sediment to the tank after centrifuging, thus separating and controlling the HRT and SRT. However, the use of centrifuges is not economical in practical terms. Anaerobic membrane bioreactors (AnMBRs) have recently evinced promise as viable alternatives to conventional anaerobic digesters for the treatment of organic waste. The membrane separation in AnMBRs decouples the SRT and HRT, enabling operations at longer SRTs [10], [11], [12], [13]. Problems involving the loss of bacteria can hence be effectively resolved. The membrane can treat food waste in the anaerobic reactor and can also be used for the treatment of organic sludge. Cheng et al. (2018) were able to operate the AnMBR system at a higher load by adding a membrane unit reactor after the continuous stirred tank reactor. However, their design required two reactors, bioreactor which needs mixing and membrane separation tank which needs membrane washing by biogas circulation. Further, Amha et al. (2019) employed a flat-sheet membrane and were able to actualize the former project using a single reactor. Despite improvements, this process required the circulation of biogas to flush the membrane and must be mandated the use of an impeller to mix the reactor. The flushing of the hollow fiber membrane only required one biogas spout underneath, while the flushing of the flat-sheet membranes needed multiple spouts to be positioned beneath the membrane. Thus, hollow fiber membranes were also found to be suitable for small reactors. In this MethodsX, the authors of this paper were able to actualize the abovementioned project using a single reactor. The proposed method allows the circulation of biogas to simultaneously flush the hollow fiber membrane and enable the mixing of the reactor; hence, this design makes system simplify and reduces operational difficulties. The current study also posits a method of monitoring the biogas production rate using a real-time gas meter.

## Substrate (Artificial food waste)

Artificial food waste (AFW) was created using boiled rice (300 g), cabbage (90 g), carrots (90 g), chicken (20 g), and small dried sardines (48 g). AFW components were measured wet, and the chicken and dried sardines were weighed after boiling. The AFW's material weight ratios were estimated based on a survey conducted in the Osaka Institute of Technology's cafeteria [1], [2], [3], [4]. The mixtures were then further homogenized into a paste using a Grindmix GM 200 grinder (Grindmix, Retsch, Haan, Germany) without a sterilization process. The AFW's total solids (TS) content was adjusted to 100 g L^−1^ by adding distilled water purged with nitrogen gas. The average AFW composition was 98 g L^−1^ of volatile solids (VS) and 46 g/L of total organic carbon. Additionally, lack of trace minerals in substrates has been reported to severely limit the growth and metabolism of hydrogenotrophic and acetoclastic methanogens [14,15]. This may lead to the accumulation of volatile fatty acids (VFAs), which play an inhibitory role for many of the organisms involved in the production of biogas [16]. Therefore, a solution of trace minerals dissolved in water was simultaneously added—100 mg of Fe as FeCl_3_⋅6H_2_O, 10 mg of Co as CoCl_2_, and 10 mg of Ni as NiCl_2_ (per 1 L of AFW) [4,5].

## AnMBR system setup and operation

**Fig. 1:**
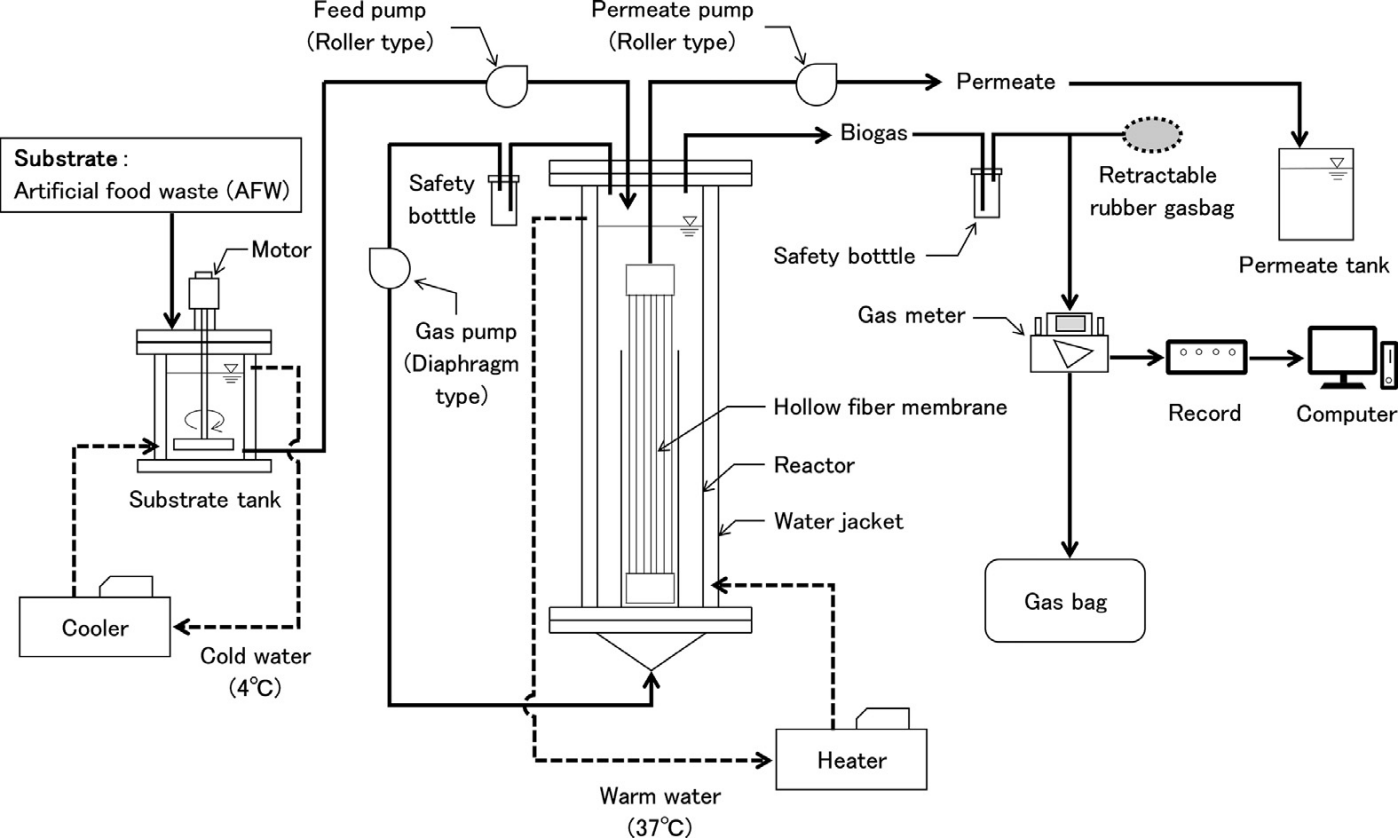
Experimental apparatus assemblage.

**Fig. 2:**
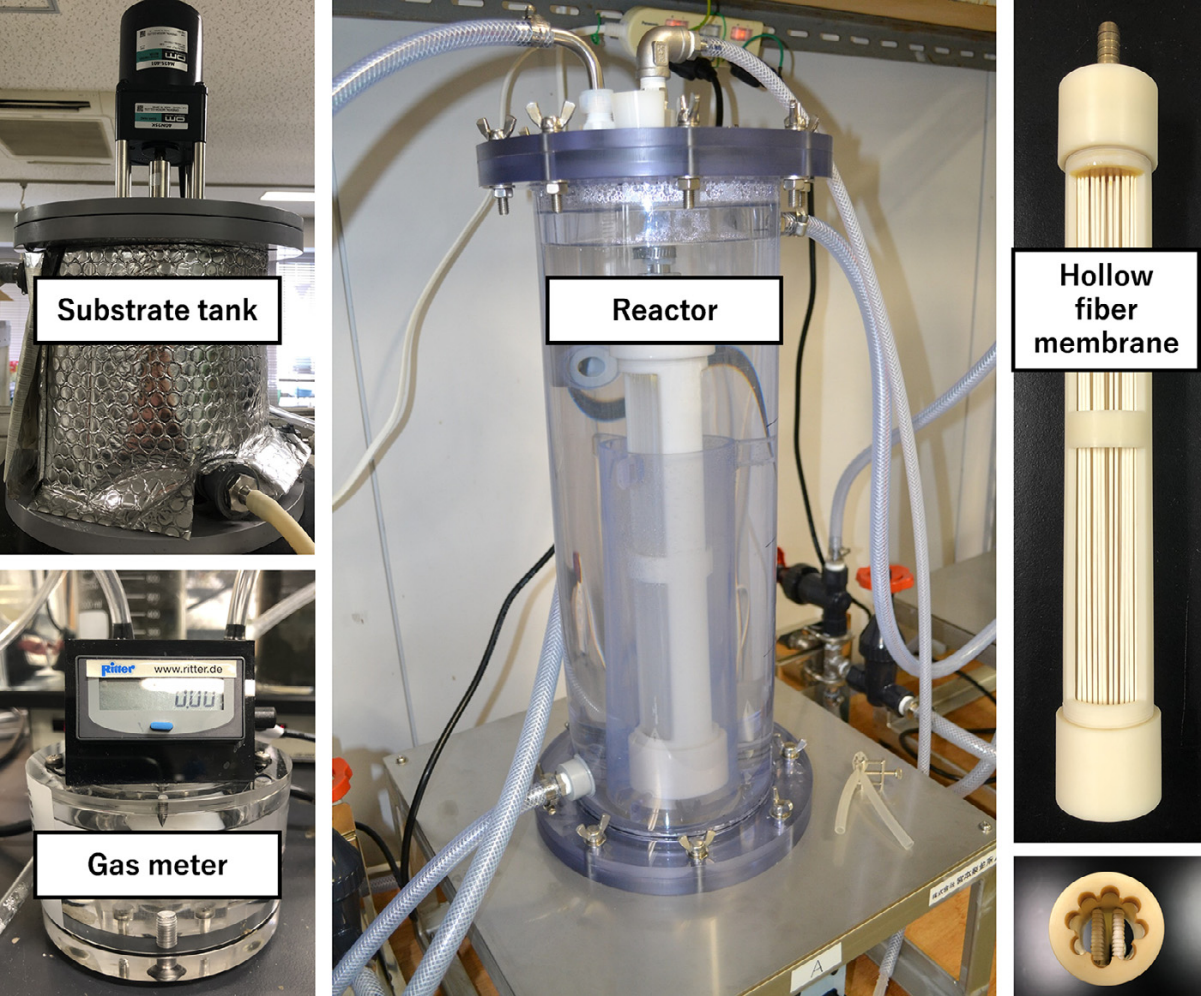
Main experimental equipment.

**Fig. 3:**
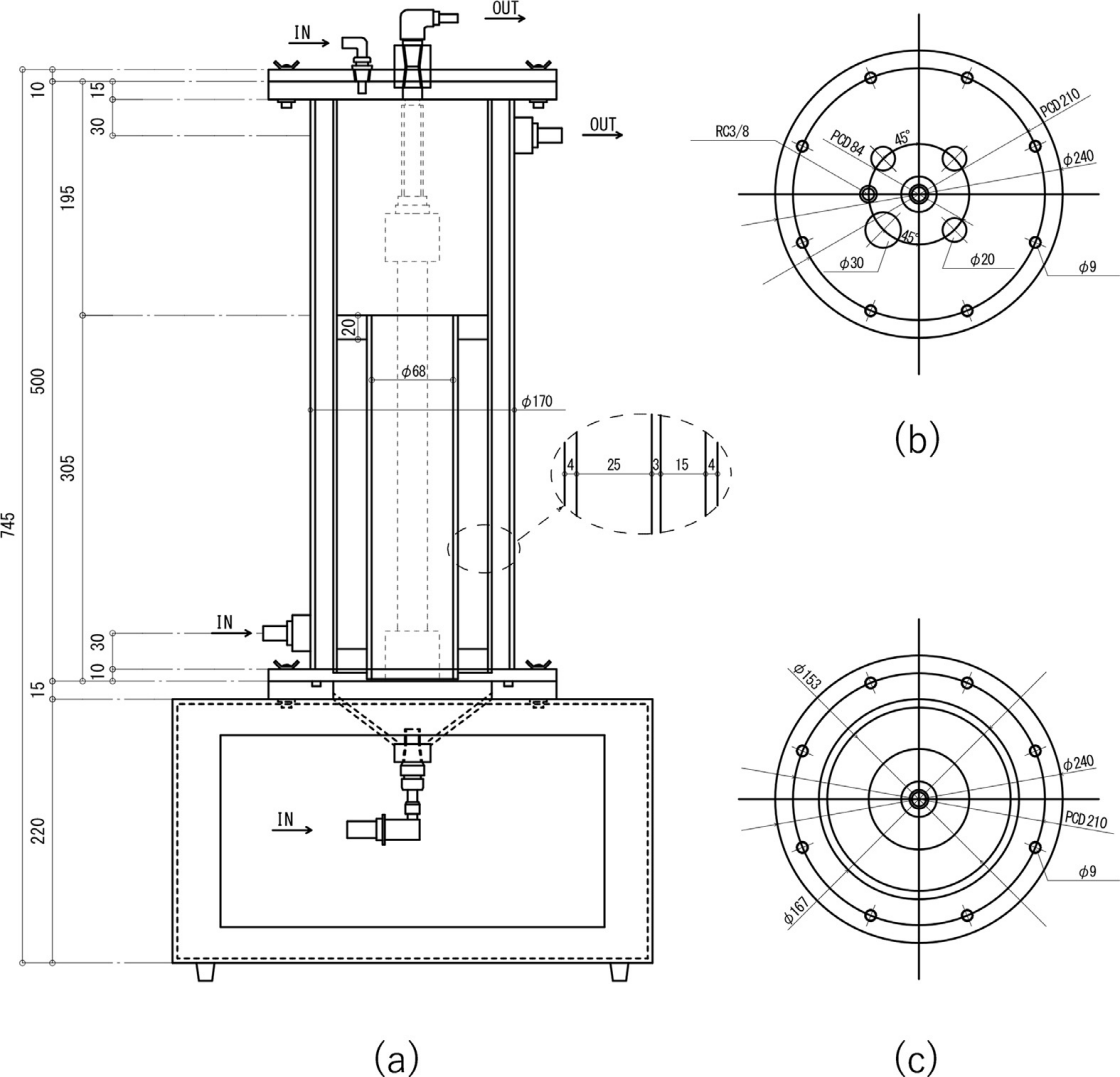
(a) Reactor longitudinal section, (b) constructive details of the reactor cap, (c) constructive details of the reactor bottom.

Figs. 1 and 2 illustrate the AnMBR system in this study, and Fig. 3 shows details of the reactor unit. The reactor comprised two cylindrical-shaped devices, 500 mm in height. The internal and external diameters, respectively, equal 126 and 170 mm, and a water jacket (width 15 mm) is sandwiched between the two. Circular bushings in the internal reactor support the membrane module and prevent its shaking due to biogas wash. The internal reactor's volume was 6 L, with an effective work volume of 4.5 L, and 1.5 L of headspace was reserved. To avoid corrosion by hydrogen sulfide gas, the reaction vessel is made of polymethyl methacrylate. A hollow fiber membrane (LSPMW-02, Sumitomo Electric Industries, Japan) used in system has an average pore diameter of 0.2 μm and an effective filtration area of 0.1 m^2^ of polytetrafluoroethylene (PTFE). The membrane module was immersed in the reaction vessel, the temperature was maintained at 37 °C with a water jacket, and the substrate tank was maintained at 4 °C, also with a water jacket. These two water jackets use, respectively, cold and hot water circulation devices. The substrate was stored in the substrate tank with an effective work volume of 2 L. To prevent the substrate's precipitation, the mixture was stirred at 60 rpm. Substrate was supplied from the substrate tank to the reaction tank via roller pumps, operated at a cycle of 1 min work and 239 min off; treated water was permeated from the membrane module to the treated permeate tank using a roller pump. Before substrate was supplied, most of the biodegradation had been completed. Thus, the roller pump was operated for 60 min before the substrate was supplied.

The initial trial operation used the internal liquid circulation method. Due to the digested sludge's high viscosity, stirring in the reaction tank was uneven, and the membrane module could not be washed well. Therefore, the membrane was washed by circulating biogas from the headspace to the bottom of the reactor with a diaphragm gas pump (085LV-1, IWAKI, Japan). Safety bottles were installed before the gas pump and the gas meter to prevent sludge from flowing into them. The use of the diaphragm gas pump in this study caused the pressure of biogas to fluctuate, raising the potential of error in the gas meter. Therefore, a retractable rubber gasbag (113401, DEBIKA, Japan) was installed before the biogas entered the gas meter so that generated biogas passed through the gas meter at a constant speed. The fermentation reactor was purged with O_2_-free N_2_ for 15 min before addition of seed sludge, which was collected from the mesophilic anaerobic digester at the sewage treatment plant and the FW digester. Table 1 displays step-by-step operating conditions during the acclimatization period of increasing the load for 80 days; it was confirmed that the gas generation amount and pH were in a steady state, and they were used for the formal experiment.

**Table 1:** The operating conditions during the acclimatization period.

OLR (g-COD L^−1^ d^−1^)	2.9	3.7	4.4	5.3	6.6
Duration (day)	1―12	13―17	18―26	27―45	46―80
HRT (day)	45	36	30	25	20
TS (g/L)	30.8	32.7	36.0	47.0	44.3
AFW add frequency (min/times)	360	300	240	240	240

In the formal experiment, the AnMBR system was operated at three OLRs, 6.6, 8.8, and 10.5 g-chemical oxygen demand (COD) L^−1^ d^−1^ to increase the load by increasing feeding volume. The corresponding HRT ranged from 20 to 12.5 days.

## Analytical methods

TS and VS were analyzed by standard methods (American Public Health Association, 2005). The AFW's total carbon was analyzed using combustion catalytic oxidation and a non-dispersive infrared (NDIR) method (SSM-5000A, Shimadzu, Japan). Biogas from the AnMBR was collected with a gas bag and quantified by thermal conductivity detection-gas chromatography (GC14B, Shimadzu, Japan) using a ShinCarbon ST 50/80 column (Shinwa Chemical Industries, Kyoto, Japan) with argon as the carrier gas (50 mL/min). In total, 500 μL of biogas were injected into the gas chromatograph, with injector, column, and thermal conductivity detector (TCD) temperatures at 200 °C, 40–200 °C, and 200 °C, respectively. Analysis of COD used a spectrophotometer (DR900, HACH, USA) and CODcr reagent (HACH 4236, HACH, USA). More details are available in our previous study [5].

## Method validation

Table 2 shows HRT, SRT, and average fluxes at different operating loads. In the existing anaerobic digestion operation, HRT and SRT are the same, but in this experiment, SRT is about three times that of HRT under different loads. This shows that use of AnMBRs effectively prevents washout of slow-growing methane-forming anaerobic microorganisms and helps maintain microorganisms at a high population level, allowing substrate to be effectively degraded. This table displays a selected part of the experimental data, and all the data can refer to existing research [5].

**Table 2:** HRT, SRT, and average fluxes at different operating loads.

	Phase I	Phase II	Phase Ⅲ
Duration (day)	81―122	123―136	137―159
OLR (g-COD L^−1^ d^−1^)	6.6	8.8	10.5
HRT (day)	20	15	12.5
SRT (day)	63	53	33
TS (g/L)	35.5	41.3	43.1→ 48.0
Average flux (m/day)	0.0011	0.0017	0.0018
AFW add frequency (min/times)	240	240	240
Operation feasibility	Enable	Enable	Failure

**Fig. 4:**
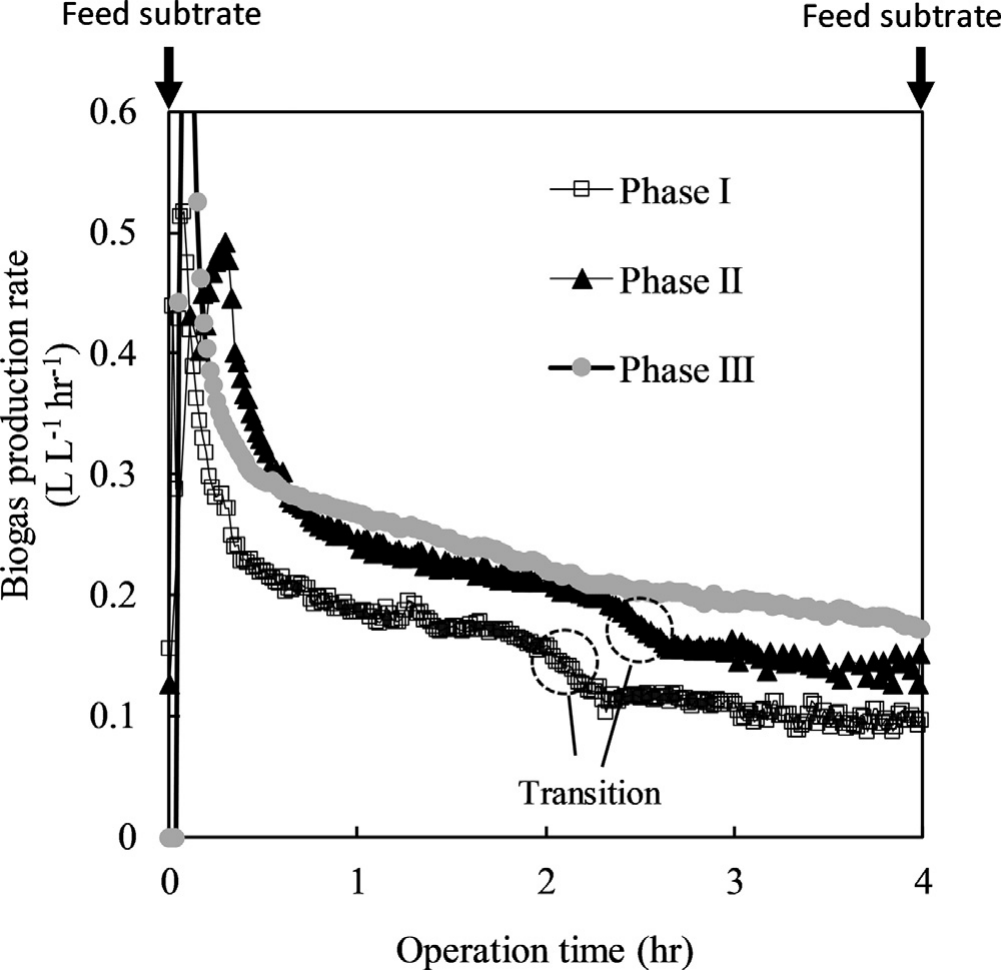
Biogas production rate (y-axis) and Operation time (x-axis). Hollow square shows Phase I (OLR= 6.6 g-COD L^−1^ d^−1^), solid triangle signifies Phase II (OLR= 8.8 g-COD L^−1^ d^−1^) and Filled circle Phase Ⅲ (OLR=10.5 g-COD L^−1^ d^−1^). Substrate was fed every 4 hr starting at 0 hr.

Fig. 4 shows the biogas production rate (y-axis) and operation time (x-axis). The hollow square shows Phase I (OLR = 6.6 g-COD L^–1^ d^–1^), the solid triangle signifies Phase II (OLR = 8.8 g-COD L^–1^ d^–1^), and the filled circle Phase III (OLR = 10.5 g-COD L^–1^ d^–1^). Substrate was fed every 4 h starting at 0 h. Analysis of results of gas meter data revealed significant transitions in the second hour of Phases I and III, showing constantly high biogas production transition to constant low biogas production. These results followed previous research [17] in which most biodegradation had been completed. However, Phase III showed no obvious transition, indicating that this phase's biodegradation was not smooth. The final experimental results also showed that Phase III could not run for a long time under such a high load. These results showed that the real-time gas meter monitoring the experiment was effective and that this monitoring method was equally effective in AnMBR. We also expect to perform various analyses using gas meter data.

Data analysis made it possible to prove that the AnMBR system was stable and that the experiment was feasible. The authors hope the design will guide researchers to experiment with AnMBR, especially for monitoring methane production rates—with appropriate adjustment wherever necessary.

In the end, the author found certain design shortcomings. The used biogas cycle for washing the membrane module generates a certain amount of foam, but too much foam would block the biogas pump. To remedy this shortcoming, the author proposes increasing the headspace.

## Declaration of Competing Interest

There are no conflicts of interest to declare.
